# Supplementary material for: Are carbon emissions trading and green financial instruments synergistic? -Comprehensive quantitative research based on content analysis
Source: PLoS One. 2024 Mar 7;19(3):e0298601. doi: 10.1371/journal.pone.0298601 (PMC10919601; doi:10.1371/journal.pone.0298601)
Supplement: S1 Data — (ZIP) [file pone.0298601.s001.zip › Data/地级市Z_F工作报告.zip!/▒▒╛⌐/└┤╫╘╣1⁄2╓┌║┼í╛┬φ┐╦╩2╛▌═°í┐_2010.docx]

——2010年1月25日在北京市第十三届人民代表大会第三次会议上

北京市市长 郭金龙

各位代表：

　　现在，我代表北京市人民政府，向大会报告政府工作，请予审议，并请市政协各位委员提出意见。

　　一、过去一年工作回顾

　　2009年是首都发展历程中很不平凡的一年。在党中央、国务院和中共北京市委的坚强领导下，全市人民坚定信心，团结奋斗，克服困难，开拓创新，较好地完成了“打赢一场硬仗、办好一件大事”的目标任务。

　　——应对国际金融危机冲击取得显著成效。初步核算，全市地区生产总值达到11865.9亿元，比2008年增长10.1%，人均地区生产总值超过1万美元；完成地方财政收入2026.8亿元，增长10.3%。城镇登记失业率为1.44%。城镇居民人均可支配收入26738元，农民人均纯收入11986元，实际增长9.7%和13.4%。

　　——新中国成立60周年庆祝活动圆满成功。实现了“隆重、喜庆、节俭、祥和”的总要求，赢得了国内外的高度评价和广泛赞誉，向党中央、国务院和全国人民交上了一份合格的答卷。

　　一年来，我们认真执行市十三届人大二次会议决议，主要做了以下几个方面工作。

　　(一)全力以赴筹办国庆活动。

　　筹办新中国成立60周年庆祝活动，是继北京奥运会、残奥会之后，中央交给北京市的又一重大光荣任务。在中央国庆领导小组和市筹委会的领导下，充分运用奥运经验，健全组织指挥体系和运行调度机制，保证了各项工作协调推进、胜利完成。

　　精心组织庆祝活动。各指挥部全面建立“双进入、双责任”工作体制，精心设计活动方案，科学安排训练演练，实现了“高质量、有创新”的目标。组织10万名群众参加国庆游行、8万名学生进行广场背景表演。运用高科技手段，突破传统表现形式，奉献了一场创意新颖、主题鲜明的联欢晚会。组建预备役、女民兵方队，代表全国民兵预备役部队接受检阅。开展丰富多彩的游园活动，成功举办第七届中国花卉博览会。依托现行管理体制，统筹指挥调度，实现了现场运行零差错、活动内外零事故。广大参演人员刻苦训练，涌现出许多感人事迹，充分表达了对伟大祖国的无比热爱。

　　全面做好服务保障。坚持“贴近服务、融入服务、主动服务”，及时帮助受阅部队解决遇到的困难。构建多层次、全方位、无缝隙的安保网络，确保了国庆活动的绝对安全。优化交通运输组织方式，实现了庆祝活动交通与社会交通的和谐运转。全力做好气象、电力、水务、环卫、通信、医疗、食品供应等保障工作，城市运行平稳有序。高质量完成长安街大修和沿线改造工程，认真做好环境整治和景观建设，城乡环境干净整洁、优美靓丽。

　　营造文明和谐社会氛围。广泛开展“迎国庆、讲文明、树新风”活动，组织“百姓宣讲”等爱国主义主题教育活动，举办130余台、240余场文艺演出。新闻宣传浓墨重彩，唱响了时代主旋律，全景展示了庆典盛况。95万名志愿者积极参与，在游园、治安、交通等领域提供了热情周到的服务。提前公布交通限行等措施，注意减少筹办工作对居民生活的影响。全市人民服务大局、全力支持、争做贡献，凝聚成办好国庆活动的强大合力。

　　(二)力保首都经济平稳较快发展。

　　2009年年初，受国际金融危机严重冲击，经济下行态势明显，保增长压力很大。在非常困难的情况下，我们坚决贯彻落实中央一揽子计划，结合实际制定实施一系列具体措施，扭转了经济增速下滑的不利局面，完成了年度各项主要经济指标。

　　促进投资快速增长。一是努力发挥政府投资带动作用，统筹安排、快速落实市政府投资309亿元，实行重大项目投资责任制，加快推进项目落地。二是创新工作思路，大规模实施政府土地储备，完成土地一级开发投资1165亿元，为调控土地供应、改善城乡环境、促进项目建设奠定了良好基础。三是创建绿色审批通道机制，高效率审批通过1040个重点项目，审批时间缩短50%左右，完成投资占全市投资总量的37.5%。四是创新拆迁安置补偿办法，引入市场价格评估机制，加大实物补偿力度，全力协调解决征地拆迁等难点问题。五是加快投融资平台建设，组建市国有资本经营管理中心，设立农业担保公司、高端制造业担保代偿资金，进一步发挥专业投融资平台作用。全社会固定资产投资在5月份结束了连续9个月的负增长，全年完成4858.4亿元，增长26.2%。

　　保持消费市场繁荣发展。贯彻落实国家促进消费各项政策措施，支付补贴资金3.7亿元，积极做好家电汽车下乡和以旧换新工作。商品住宅销售面积增长82.3%，机动车销售量增长30.8%。广泛开展各类主题促销活动，推进特色商业街区改造。加快农村流通市场建设，“一乡一集市”覆盖率达到72%。实施奥运后旅游发展战略，旅游总收入达到2442.1亿元，增长10%。全年完成社会消费品零售额5309.9亿元，增长15.7%。

　　推动对外经贸稳定发展。面对国际市场需求萎缩的严峻形势，明确提出“位次不降低、份额不减少”的工作目标，增加外贸发展资金，支持企业稳定拓展国际市场。地区进出口总额降幅逐步收窄，出口达到483.6亿美元，保持了在全国的份额。制定实施促进服务贸易、服务外包发展政策，加快建设服务外包示范城市。天竺综合保税区封关运行。制定实施跨国公司地区总部政策，完善投资促进工作机制，新设立投资性公司15家，实现了合同外资、实际利用外资双增长，新增境外投资企业140家。加强京港、京澳、京台交流，成功举办科博会、文博会、服务贸易大会和国际版权博览会。

　　加大企业帮扶力度。坚持运营帮扶与发展帮扶并举，及时制定实施66项帮扶措施。通过降低社会保险缴费费率、调整缴费周期等措施，使企业和职工群众受益97.3亿元。发放岗位补贴和社会保险补贴5.5亿元，帮助企业稳定7.3万个就业岗位。多渠道安排资金50亿元，支持企业稳定生产经营。落实结构性减税政策，减轻企业和居民负担171.5亿元。成立帮扶机构，协调解决企业反映的困难和问题1594项。重视发挥各类金融机构的作用，采取多项工商管理措施，帮助企业解决融资难题。全市企业生产经营基本保持稳定，一批骨干企业逆势增资扩产，规模以上工业增加值增长9.1%。

　　高度重视支援什邡恢复重建工作，落实“三年任务两年完成”的要求，累计拨付援建资金52.6亿元，完成80%的援建实物工程量。

　　(三)大力促进经济结构调整。

　　坚持保增长与调结构相结合，发布实施六大产业调整振兴规划，进一步增强了首都发展的协调性和可持续性。

　　加快推进科技创新。制定实施中关村国家自主创新示范区建设意见，着力推进先行先试改革，202家单位申报开展股权激励试点，核心区行政审批制度改革下放17项市级审批事项。整合首都创新资源，搭建生物医药、新材料等4个领域平台，与中央单位合作建立12个研发实验服务基地，推动成立30个产业技术联盟。积极开展高新技术企业认定辅导工作，采购自主创新产品33.1亿元，9家科技企业在创业板成功上市。完善知识产权服务体系，全市专利授权量增长29.1%，技术合同成交额增长20.4%。积极支持未来科技城建设，实施海外人才聚集工程、中关村高端领军人才聚集工程，人才引进工作取得新进展。

　　大力发展高端产业。加强与中央部委和企事业单位合作，与24家单位签订战略合作协议，并设立综合服务平台，积极做好对接服务。认真抓好京东方八代线、中航工业园等重大产业项目落地，积极推动石化新材料、数字电视、移动硅谷等产业基地建设。组织实施飞思卡尔通讯公司、德尔福汽车配件和萨博汽车技术等并购项目。研究制定金融业、软件和信息服务业发展意见，加快丽泽金融商务区建设，启动商务中心区、金融街扩区工作。投入资金13亿元，支持文化创意、体育、旅游等产业发展。完善促进影视动画、网络游戏等产业发展的政策措施，成立文化创意产业联盟。生产性服务业、文化创意产业分别增长9.8%和11.2%，第三产业比重达到75.8%。积极做好节能减排各项工作。强化机动车污染控制，更新淘汰黄标车10.6万辆，支持组建货运“绿色车队”，采购1000辆节能环保公交车。完成城镇建筑节能改造392万平方米、农民住宅增温节能改造1.6万户。开展20蒸吨以上燃煤锅炉清洁能源改造试点，完成179个老旧小区供热管网改造、10万户居民老楼通气工程，文保区平房“煤改电”全面完成。推广高效照明产品1370万只。新增节水灌溉面积10万亩，建设节水型单位和小区166个。淘汰“三高”企业62家。节能减排工作继续走在全国前列。

　　促进城乡一体化发展。出台促进城市南部地区加快发展行动计划和永定河绿色生态发展带规划，认真落实新城建设实施意见和生态涵养发展区发展意见。北坞、大望京村城乡一体化改革试点取得突破性进展。制定实施都市型现代农业基础建设及综合开发规划，新增设施农业4.3万亩。出台农产品加工业发展指导意见，实施乡村旅游改造提升工程，农业观光园和民俗旅游进一步发展。制定小城镇建设发展规划和实施意见，新编制完成村庄建设规划1674个。筹集80多亿元支持农村“5+3”工程建设，覆盖1700多个行政村，惠及120万农村居民。搬迁山区农民6811人。废弃矿山修复、生态清洁小流域治理深入推进。

　　(四)加快重点领域和关键环节改革。

　　加大行政管理体制改革力度。完成市与区县政府机构改革，调整优化19个市政府工作部门，减少市级议事协调机构和临时机构27个。提出“加快、简化、下放、取消、协调”的要求，深化行政审批制度改革，各部门共取消审批事项24项、下放行政审批权限103项。

　　积极推进农村各项改革。建立市和区县两级农地流转信息平台，开展集体林权制度改革试点。全面落实各项农业补贴政策，建立低收入农户增收帮扶机制。346个单位完成乡村集体经济产权制度改革。制定农民专业合作社法实施办法，新注册合作社1270家。

　　大力推动金融改革创新。启动农村金融综合改革试验区建设，设立农业产业投资基金，建立了农业再保险机制。批准成立21家小额贷款公司、4家村镇银行。成功争取房地产信托投资基金、消费金融公司、省级投融资平台发行债务融资试点。6家商业银行在中关村设立了服务科技型中小企业的专营机构。技术、林权、版权、粮食交易所挂牌开业，要素市场不断完善。

　　稳步推进国有企业改革。成功实施首旅集团、首农集团等六大重组项目，市国资部门直接出资企业减少至46家。完成125户劣势企业退出工作。着力推进国有企业改制上市，新增7家上市公司。国有资产监管进一步加强，市属国有企业利润增长59.5%。

　　(五)下大力气解决关系群众切身利益的实际问题。

　　加强就业和社会保障工作。出台促进高校毕业生就业的15条政策，实施大学生村官和社工计划，增设1万多个基层社会管理、公共服务和教学科研岗位，高校毕业生就业率达到96.4%。建立城乡平等的就业制度，帮扶10.8万名就业困难人员实现就业、10.2万名农村劳动力转移就业，零就业家庭实现动态脱零。建立企业裁员报告制度和调解联动机制，劳动关系保持和谐稳定。启动“社会保障卡”发放工作，192家定点医疗机构实现就医实时结算。建立城乡居民养老保险制度，率先实现养老保障制度城乡一体化。实施11项老年优待政策，新增养老床位1.58万张。出台居家养老、助残服务“九养政策”。加快实施保障性安居工程，累计配租廉租房1.9万户，修缮旧城居民房屋2.45万户，完成城镇危房解危1052户，搬迁三区三片棚户区居民8000多户。翻建维修6500户农村特困和优抚家庭住房。新开工和收购政策性住房937.9万平方米，竣工229.5万平方米。

　　加快发展社会事业。完成200所小学标准化建设，修缮300所农村中小学教师集体宿舍，启动中小学校舍安全工程。稳步实施义务教育学校教师绩效工资改革。出台加强农村教师队伍建设意见，引导优质教育资源向农村辐射。制定实施“健康北京人”十年行动规划。新农合人均筹资标准提高到420元，参合率达到95.7%。规范医疗机构管理，启动公费医疗人员基本医疗保险改革试点，推动医疗便民服务，在49家三级医院实施预约挂号。大力实施文化惠民工程，文化活动中心、数字电影厅实现行政村全覆盖，新建1020个农家书屋，有线数字电视覆盖15个区县230万户居民家庭。完成第三次文物普查实地调查，公布第三批市级非物质文化遗产名录。深入开展群众体育活动，成功举办了15项国际体育赛事。

　　不断提升城市建设管理水平。地铁4号线顺利开通，轨道交通运营里程达到228公里、在建里程280.2公里。六环路、京承高速全线贯通，高速公路总里程达到884公里。优化公交线网和换乘环境，公共交通年客运量达到65.9亿人次，公交出行比例提高到38.9%。认真落实大气污染控制措施，市区空气质量二级以上天数达到78.1%。全面推进生活垃圾处理工作，市区生活垃圾年产生量首次下降，全市生活垃圾无害化处理率达到95.8%。稳步推进北运河水系综合治理，加快污水处理厂升级改造，市区、郊区污水处理率分别达到94%和51%，利用再生水6.5亿立方米。开展生态区县、环境优美乡镇和生态村创建活动，建成19处郊野公园，推进京津风沙源治理、三北防护林建设、太行山绿化工程，全市林木绿化率达到52.6%，人均公园绿地面积达到14.5平方米。积极推动城市建设管理方式转变，城市服务管理的数字化、精细化水平进一步提高。

　　(六)坚决维护首都和谐稳定。

　　扎实推进社会建设。出台7项加强社会建设的配套政策。创新“两新”组织服务管理模式，组建650个商务楼宇社会工作站。积极发展“枢纽型”社会组织，实行社会组织设立审批“一站式”服务。在600个社区开展规范化建设试点，改善350个社区办公和服务用房条件。落实征兵新政策，实施拥军优属工程，军政、军民关系进一步巩固，驻京解放军、武警部队官兵在筹办国庆活动等工作中作出了重要贡献。人口和计划生育工作扎实开展，妇女、儿童、老龄事业健康发展。残疾人社会保障水平进一步提高，残疾人事业全面发展。深入开展民族团结进步创建活动，坚持做好宗教工作，认真落实惠侨利侨措施。继续加强法制宣传教育，大力推进公益法律服务体系建设。顺利完成第七届社区居委会换届选举，基层民主自治能力进一步增强。

　　全面推进“平安北京”建设。强化社会治安防控，坚持专群结合、群防群治，开展专项打击整治，打赢了安全稳定“三大战役”，实现了“四个坚决防止”，保持了良好的社会秩序。强化安全生产作业规范和责任体系建设，开展全市安全大检查和隐患排查整改，全力压减各类安全生产事故。完善食品、药品安全监管网络，检测抽查合格率均超过98%。深化应急管理体系建设，加强城市风险评估与控制，突发事件应急处置能力进一步提高。探索建立多元化矛盾纠纷化解体系，开展“信访积案化解年”活动，信访总量和集体访次数“双下降”。拨付8.8亿元专项资金，落实属地、部门、单位、个人四方责任，免费接种疫苗，加强重症患者救治，重视发挥中医药的作用，有效防控甲型H1N1流感疫情，保证了人心安定和社会稳定。

　　各位代表，过去一年，我们认真开展深入学习实践科学发展观活动，落实领导干部作风建设年各项部署，进一步加强了政府自身建设。市政府及组成部门全面执行市人大及其常委会决议和决定，自觉接受监督并报告工作。自觉接受政协民主监督，认真听取各民主党派、各人民团体和各界人士意见。全年共办理全国和市人大代表议案、建议1678件，政协建议案、提案1425件。加强政府法制建设，共提请市人大常委会审议地方性法规4项，制定、修订政府规章8项。完成第二次经济普查任务。投入125.6亿元，全面完成关系群众生活的57件重要实事。切实精简会议，市政府常务会、专题会减少23%，严格控制一般性支出，压缩出国、车辆购置及运行、公务接待等费用5292.8万元。实施“三效一创”绩效管理，加大督查考核力度，强化行政监察和审计监督，加强廉政建设和反腐败工作，行政效能和服务水平有了新提高。

　　各位代表，回顾一年的工作，我们深感成绩确实来之不易。这是党中央、国务院和中共北京市委坚强领导的结果，是市人大、市政协和社会各界监督支持、共同努力的结果，是各区县各部门扎实工作、全市广大人民群众团结奋斗的结果。在此，我代表市人民政府，向辛勤工作在各条战线、各个岗位上的全体市民和来自全国各地的劳动者，向人大代表、政协委员，向各民主党派、各人民团体和各界人士，向中央各部门、兄弟省区市，向驻京解放军和武警部队官兵，向所有关心支持首都建设的香港特别行政区同胞、澳门特别行政区同胞、台湾同胞、海外侨胞和国际友人，表示崇高的敬意和衷心的感谢！

　　在看到成绩的同时，我们也清醒地认识到，首都经济社会发展还存在不少困难和问题。一是国内外经济环境不确定因素很多，出口恢复增长还存在较大困难，社会投资跟进不足。二是人口资源环境矛盾十分突出，加快转变发展方式、调整经济结构的要求更加紧迫。三是城乡区域发展差距仍然较大，城乡结合部整治改造困难较多，统筹推动区域协调发展的任务艰巨。四是自主创新能力还不够强，首都科技优势还没有完全发挥，新兴产业还需要加快培育。五是影响首都科学发展的体制机制障碍仍然没有完全消除，重点领域和关键环节改革需要加快突破。六是关系群众切身利益的就业、教育、医疗、住房、交通等领域还存在不少问题，保障和改善民生需要加大力度。七是政府作风建设和效能建设要进一步深化，建设服务型政府需要下更大的功夫。对这些问题，我们一定要高度重视，采取更加有力的措施，不断加以解决。

　　二、2010年主要任务

　　当前，首都已进入了全面建设现代化国际大都市的新阶段。面对我国国情国力和国际地位的新变化，面对首都在国家工作大局中应承担的重要任务，面对推动首都科学发展、促进社会和谐的历史责任，我们必须立足当前，着眼建设世界城市，坚持高标准建设、高质量管理、高水平服务，进一步提高现代化、国际化水平，使首都的发展建设与国家和人民的要求相适应。

　　2010年是实施“十一五”规划的最后一年，也是在新的起点上全面推进“人文北京、科技北京、绿色北京”建设的重要一年。2010年发展环境可能好于2009年，但国内外形势复杂多变，经济社会发展面临新的考验。各级政府、各部门要紧紧围绕推动首都科学发展这一中心任务，进一步增强机遇意识、忧患意识和竞争意识，树立强烈的使命感、责任感和紧迫感，进一步解放思想，充分利用各方面有利条件和积极因素，深化改革创新，努力化解矛盾和困难，牢牢把握各项工作的主动权。

　　做好2010年的政府工作，要全面贯彻落实党的十七大和十七届三中全会、四中全会、中央经济工作会议、市委十届七次全会精神，深入贯彻落实科学发展观，全面实施人文北京、科技北京、绿色北京行动计划，着力抓好自主创新，加快经济发展方式转变；着力抓好一批重大工程和重大项目，加快产业结构优化升级；着力抓好改革开放，进一步增强首都经济发展动力和活力；着力抓好改善民生，使发展成果更好地惠及人民群众；着力抓好国际大都市建设，进一步提高首都现代化、国际化水平；全面完成“十一五”规划目标，努力实现首都经济社会又好又快发展，加快建设繁荣、文明、和谐、宜居的首善之区。

　　2010年全市经济社会发展的主要目标是：地区生产总值增长9%；城镇登记失业率控制在2.3%以内；城乡居民收入实际增长6%以上；居民消费价格涨幅控制在3%以内；地方财政收入增长9%；万元地区生产总值能耗、水耗均下降4%。

　　2010年要着重抓好以下七个方面的工作：

　　(一)继续把促进首都经济平稳较快发展放在首位。

　　坚持立足于扩大内需，保持各项政策措施的连续性、稳定性，不断夯实首都经济稳定向好的基础。

　　积极扩大消费需求。以增强城乡居民消费能力为重点，优化消费环境，释放消费需求，实现社会消费品零售额增长12%。一是努力增加城乡居民收入。稳步提高社会保障待遇标准，落实国家收入分配政策和“三农”各项补贴政策，健全劳动者工资正常增长和支付保障机制。二是稳定汽车和住房消费。继续做好家电汽车下乡和以旧换新工作，合理引导汽车消费。扩大保障性住房供给，引导房地产企业加快开发和入市交易，促进存量房交易。三是大力发展旅游消费。创新观念和机制，发挥历史文化资源、奥运城市优势，提升首都旅游发展水平。深化旅游市场推广和区域合作，完善旅游公共服务体系，推进旅游集散特色镇建设，引进大型项目，鼓励社会资本参与开发，努力把旅游业培育成重要支柱产业。四是繁荣郊区消费市场。引导大型商贸企业向新城拓展布局，加强郊区现代商业设施和流通网络建设，提高“一乡一集市”、连锁便民店和农资超市覆盖面。五是拓展消费新领域。积极申办高端体育赛事，扩大文化、体育、休闲、健身、养老服务等消费。推进消费金融公司试点，大力发展电子商务。支持引进国内外知名品牌和企业，加快建设国际商贸中心。

　　保持投资适度增长。落实两年政府投资1400亿元、带动社会投资1万亿元的计划，统筹安排政府投资700亿元，实现全社会固定资产投资增长11%。一是抓好重大项目建设。坚持和完善部门联动机制，发挥区县主体作用，加快投资项目审批，促进重大项目落地。二是稳定房地产投资。继续做好土地储备，完成1000亿元土地一级开发投资任务。坚持节约集约利用和“多储快供”，优化土地供应结构，推动二级开发投资稳定增长。三是提高产业投资比重。积极运用贷款贴息、专项补贴和资本金注入等方式，支持符合首都功能定位的重点产业发展，鼓励支持企业加大技改投资。四是认真做好中央单位投资项目对接服务工作，争取一批项目尽快落户北京。同时，进一步加强投资监管，重视做好债务风险防范工作。

　　努力促进民营经济和中小企业发展。发挥各类投融资平台作用，制定出台促进政策，鼓励民间资本进入基础设施、市政公用事业、金融服务、高端制造业等领域。落实国家相关政策措施，推动制定中小企业发展促进条例。建立健全中小企业信用担保风险补偿机制，逐步扩大中小企业再担保资金规模。稳步发展各类集合债、集合票据和集合信托，增加市区两级财政用于中小企业贷款贴息的投入，努力缓解中小企业融资困难。研究制定面向中小企业的财政、土地、用房等支持政策，切实减轻企业负担。支持中小企业加大研发投入、加快结构调整，提高技术创新能力和产品质量。

　　营造良好的市场环境。高度关注物价变动趋势，做好煤电油气运的运行保障和重要农副产品供给，保持市场稳定。抓好食品、药品安全监管工作，制定实施食品安全三年行动计划，完善流通环节食品安全自检体系，实施基本药物全品种检验，建立健全市场退出机制。积极培育诚信文化，加快完善企业信用信息系统，加强市场监管和治理整顿，坚决打击假冒伪劣等违法行为，维护公平公正的竞争秩序和交易环境。

　　(二)下更大决心加快转变经济发展方式。

　　进一步完善体制机制、政策导向和考核评价体系，坚持优化一产、做强二产、做大三产，狠抓“三个一批带动工程”，深入推进结构调整，不断提高经济发展的质量和水平。

　　切实抓好中关村国家自主创新示范区建设。落实国务院批复精神，加快建设具有全球影响力的科技创新中心。一是加快推进先行先试改革。研究制定示范区法规，深化股权激励、科技金融、工商管理、社会组织等改革试点，争取建设场外交易市场。进一步下放市级行政审批权限，增强核心区的管理功能。二是完善示范区管理体制。加强市级管委会的组织协调和督办落实职能，设立中关村发展集团公司，加大各分园和产业基地的统筹协调力度。完善一区多园的产业空间布局，以海淀区、昌平南部地区构成的核心区为依托，整合未来科技城、软件园、生命科学园、环保园、永丰产业基地等空间资源，加快建设北部研发服务和高新技术产业聚集区。三是鼓励企业成为创新主体。支持产业技术联盟和民营科技企业承接重大科技项目，完善科技条件平台开放共享机制，加快构建产学研相结合的技术创新体系。继续做好国家重大科技专项和重大科技基础设施对接工作，实施科技振兴产业工程和科技支撑工程，努力在重大关键技术上取得突破。制定实施创新型企业“十百千计划”。四是加大科技成果产业化支持力度。健全项目跟踪和筛选机制，整合政府产业投资，以5年不少于100亿元的规模，集中支持一批成长性好的企业和重大科技成果产业化项目。加大政府采购自主创新产品力度，全年完成40亿元采购任务。五是深化科技金融创新。积极争取更多企业在创业板上市，打造“中关村”板块。支持天使投资、创业投资、股权投资聚集发展，发挥好创业投资基金的作用，逐步完善覆盖技术创新全过程的金融服务体系。深入实施首都知识产权战略，扩大知识产权质押贷款，推动专利权交易。继续实施吸引高端人才的政策，打造人才与创新、创业高地。

　　加快重点功能区建设。推进亦庄开发区和大兴区行政资源整合，理顺管理体制，拓展发展空间，抓好一批重大项目，培育数字显示、移动通信、新能源等产业集群。发挥亦庄开发区的带动作用，推动丰台科技园、大兴生物医药产业基地、通州光机电一体化基地等联动发展，建设南部高技术制造业、战略性新兴产业聚集区。全面推进金融街、商务中心区、丽泽金融商务区建设，加快建设人民银行结算中心、征信中心等金融后台项目，建立文化产权、矿业权、碳交易、存量房交易等要素市场，吸引各类金融机构在京发展。加快实施首钢厂区调整建设规划，发展高端替代产业，带动西部地区发展。发挥天竺综合保税区作用，加快临空经济区发展。认真落实规划，积极推进马坊、马驹桥、良乡等物流基地建设。做好石化新材料、新能源汽车和高端汽车零部件等特色产业基地建设和项目引进工作。

　　大力发展战略性新兴产业。进一步细化方案，分解落实责任，实施重点产业调整振兴规划，着力引进建设一批产业项目。加快新能源汽车产业化，努力开发纯电动乘用车，发展混合动力客车和商务车。积极发展风能、太阳能等可再生能源产业，推动延庆智能电网示范区、平谷绿色能源产业基地建设。大力发展生物医药产业，努力形成以创新药物研发和先进医疗设备制造为龙头的产业链条。积极推动中科院科教园区发展，继续支持中航工业园、北京现代汽车三工厂等一批制造业项目建设。以市场拓展为重点，组建北京软件和信息服务交易中心，发展壮大信息服务业。推进“感知北京”示范建设，积极发展物联网产业。完善融资服务体系，加快推进国家级动漫游戏产业园区、新媒体产业基地、出版产业园、文化娱乐主题公园和演艺中心等一批重大项目建设，做大做强文化创意产业。

　　深入推进节能减排。积极发展低碳经济，全力打造绿色生产体系、消费体系和环境体系。严格执行建筑节能设计标准，实施500万平方米城镇住宅、2.2万户农民住宅节能改造。继续淘汰黄标车，采购1000辆纯电动环卫车和200辆节能环保公交车。推广高效照明产品1200万只，推进燃煤锅炉清洁能源改造，完成1000蒸吨改造任务。办好“节能超市”，健全节能环保产品推广激励机制，鼓励企业采用节能新技术、新工艺、新设备、新材料，提高清洁能源的使用比例。实施污染物总量控制，开展排污权交易改革试点。研究建立循环经济评价指标体系，大力推进清洁生产，扩大循环经济试点。加强宣传教育，营造节约资源能源、保护环境的良好社会氛围。

　　统筹谋划首都科学发展的新蓝图。坚持协调发展、创新发展、绿色发展、共享发展原则，深化对事关首都发展转型重大问题研究，集中各方面智慧，组织编制“十二五”规划纲要及各项专业规划，加强对各区县发展规划编制工作的统筹指导。

　　(三)加大统筹城乡区域发展力度。

　　进一步强化强农惠农政策，有针对性地解决区域发展中的突出矛盾和问题，促进城乡一体化和区域协调发展。

　　加快农村改革发展步伐。坚持最严格的耕地保护制度，加大土地执法力度。完善土地承包经营权流转支持保护政策，抓好集体土地所有权和建设用地使用权确权发证工作。积极推进集体林权制度改革，实施公益林生态效益补偿，加强林业碳汇工作。加快农村金融综合改革试验区建设，深化村镇银行、农村资金互助社和小额贷款公司试点，扩大政策性农业保险覆盖面。深化农村综合改革。加快乡村集体经济产权制度改革步伐，支持农民专业合作社发展。健全基层农技推广等公共服务体系，制定实施提升农产品质量安全水平三年行动计划。完成30万亩农田基本建设任务，新增设施农业4万亩，着力推动籽种、苗木花卉等都市型现代农业发展。积极发展农产品加工业和特色手工业。规划建设高水准的农产品物流体系。继续推进低收入农户“共同致富行动计划”，促进低收入农户增收。做好世界草莓大会、种子大会和中国国际园林博览会筹备工作。安排转移支付24.8亿元，全面完成新农村建设“5+3”工程。加快小城镇建设，以42个重点镇为龙头，以产业园区和农民就业基地为平台，大力发展镇域经济，推动郊区城镇化。

　　加快城乡结合部改革发展。总结推广北坞村、大望京村和旧宫镇的成功经验，整体启动50个重点村的城乡一体化建设，同步推进“城中村”改造。坚持政府主导、农民主体、部门联动、社会参与，强化区县政府的主体责任和综合协调作用，鼓励各地结合实际大胆探索。把改善农民居住条件、发展产业和稳定农民就业放在突出位置，加大基础设施、公共服务设施建设和管理力度，积极发展适宜产业，落实绿化隔离地区政策，推动三海子等郊野公园建设。探索补偿安置新模式，积极引入城市社会管理和公共服务机制。加快农村集体资产处置和产权制度改革，做大做强集体经济，努力实现农民利益得保护、就业有保障、增收有途径。

　　加快新城建设。完善新城外部交通和内部路网，集中力量建设一批水电气热和垃圾处理等配套设施。推进新城滨河森林公园、生态走廊建设，初步形成新城外部绿化带。完善产业服务配套设施，引导教育、卫生等优质公共资源向新城拓展，增强对产业和人口的吸引力。着力抓好功能组团开发，高水平启动重点功能区建设。2010年要集中力量，聚焦通州，借助国内外资源，按照世界一流水平高起点谋划，实实在在地启动一批项目，努力取得重大突破，发挥好示范带动作用。

　　加快实施城南行动计划。以基础设施建设为重点，三年投入2900亿元，统筹实施一批交通、市政、公共服务项目和危改、能源安居工程，改善城市南部、西南部地区发展环境。按照“一轴一带多园区”发展格局，积极推进重点产业园区建设，大力吸引高端产业项目落户，提升产业发展水平。加快永定河绿色生态发展带建设，完成控制性详规编制，推进世界地质公园建设，实施一批河道治理、生态建设和产业项目。

　　加快生态涵养发展区建设。启动山区500公里主线、400公里支线道路建设，完成5000名山区农民搬迁任务。加快落实沟域发展规划，制定促进扶持政策，抓好7条沟域的国际招标，鼓励社会力量参与沟域经济发展。大力培育生态服务型经济，继续推动区县合作产业共建基地建设，支持煤矿、非煤矿山关停地区替代产业发展，做好人口疏解、农民转移就业工作，引导先进生产要素向生态涵养发展区转移。搞好重点生态工程建设，加强生态清洁小流域和废弃矿山综合治理，推进宜林荒山绿化和低效生态林改造，不断提高生态建设水平。

　　(四)努力提高城市可持续发展水平。

　　进一步加大工作力度，大胆探索创新，努力破解城市发展难题。

　　深入实施公交优先发展战略。着力构建以轨道交通为骨干、地面公交为主体、多种交通方式协调运转的绿色出行系统，力争公交出行比例达到40%。积极推进12条轨道交通建设，力争亦庄线、大兴线以及房山线、昌平线、顺义线一期建成通车，运营总里程超过300公里，提高既有线路运力和安全服务水平。优化调整公交线路，扩大公交专用道系统，加快综合客运枢纽、公交场站和交通集散配套工程建设，完成蒲黄榆路等4条城市快速路建设任务，全面启动重点镇的高速公路联络线建设。落实第七阶段缓解市区交通拥堵措施，抓好停车基础设施建设，实施差别化停车收费政策，深入整顿停车秩序。建立交通运行协调指挥中心，完善地面公交智能调度系统，提高交通运行综合协调指挥和应急保障能力。

　　加大污染防治力度。坚持“增能力、调结构、促减量”，提高生活垃圾减量化、资源化、无害化水平。做好大型生活垃圾循环经济园区选址规划，加快推进垃圾无害化焚烧处理，全面推行餐厨、果蔬、园林垃圾资源化处理，开展生活垃圾“零废弃”管理试点，加强非正规垃圾填埋场治理。完善分类收集运输体系，新建改扩建居住小区、社会单位全部实行生活垃圾和餐厨垃圾分类处理。健全管理体制，探索垃圾收费改革。强化对危险废物产生和经营单位、涉源单位的监管。加快北运河水系综合治理，推进垡头、沙河等污水处理厂建设，用三年时间完成中心城区污水处理厂升级改造。加强污水和再生水管网建设，完成定福庄至高碑店污水调水工程，提高污水处理和再生水利用水平。认真落实第十六阶段控制大气污染措施，力争二级以上天数达到73%。

　　加强资源能源保障。实行最严格的水资源管理制度，从战略高度研究建设节水型城市。认真落实各项节水措施，开展阶梯式水价试点。建设新应急水源，加强水源地保护，启动潮白河水资源循环利用工程，加快南水北调市内配套工程建设，实施供水管网改造，确保城乡用水安全。基本完成远郊区县33座区域集中供热中心及配套管网建设，新增集中供热300万平方米，同时选择合适方式解决区域供热问题。建成陕京三线天然气进京工程，建设六环路天然气管线系统，推进远郊区县燃气主干管线建设，启动重点镇燃气工程，解决中心城区5万户居民老楼通气问题。深入开展地下管线隐患排查整改，建立运行监控管理长效机制，确保安全运行。

　　完善城市服务管理。认真做好第六次人口普查，研究人口规模、结构与布局问题，探索建立综合调控机制，通过城市功能疏解、产业结构升级和布局调整，促进人口有序迁移与合理分布。加强出租房屋管理，创新流动人口服务。健全城市管理综合执法长效机制，进一步改善首都环境秩序。规范夜景照明、户外广告、牌匾标识、架空线等管理，着力改善老旧小区、街巷胡同、平房区、城乡结合部、农村地区的居住环境。推动物业管理纳入社区建设，研究引入第三方监管机制，促进物业管理行业健康有序发展。加快信息基础设施建设，提升互联网宽带接入标准，大力发展3G网络。完善城市运行信息管理和公共服务平台，提高网格化管理水平。建立城市风险管理长效机制，加强应急保障体系建设，强化基层应急管理，提高应对自然灾害、突发公共安全事件能力。

　　(五)更加重视保障和改善民生。

　　大力发展以改善民生为重点的社会事业，继续办好直接关系群众生活的重要实事，让人民群众得到更多实惠。

　　千方百计稳定扩大就业。实施更加积极的就业政策，探索建立统一的人力资源市场和公共就业服务平台，实现城镇新增就业36万人。继续实施大学生村官和社工计划，完善就业指导服务和见习制度，切实做好高校毕业生就业工作，促进青年失业群体就业。开发多种形式的公益性岗位，帮助9万名城镇就业困难人员实现就业，动态消除零就业家庭。将高失业率地区就业倾斜政策延伸到生态涵养发展区，深化城乡就业协作，建立纯农就业家庭就业援助制度，帮助7万名农村劳动力转移就业。整合各类扶持政策和资源，促进创业带动就业。统筹城乡职业培训体系，鼓励企业开展职工在岗培训，加快主导产业、新兴产业专业化人才队伍建设。培训家政服务、医院护理和养老护理专门服务人才2万人。全面推进劳动合同制度，维护劳动关系和谐稳定。

　　加快建立城乡统一的社会保障体系。整合“新农合”、“一老一小”、无业居民大病医疗保险制度，推动居民医疗保障制度城乡一体化。完善医保惠民政策，扩大报销范围，提高报销水平，进一步减轻群众医疗负担。深化公费医疗制度改革，将区县公费医疗人员全部纳入基本医疗保险体系。全面推广“社会保障卡”，全市定点医疗机构实现“持卡就医、实时结算”。做好养老保险关系转移接续工作。牢固树立“大民政”理念，统筹城乡社会救助制度，缓解低收入群体生活困难，推动社会福利由补缺型向适度普惠型转变。认真落实“九养政策”，进一步完善老年优待措施。实现优抚对象抚恤补助标准城乡并轨。加大慈善公益事业扶持力度。

　　促进教育事业协调发展。坚持推进教育公平，义务教育新增经费继续向农村地区、薄弱学校倾斜。深入实施校舍安全工程，支持300所中小学楼房校舍抗震加固改造。提高公办学校接收来京务工人员子女能力，规范打工子弟学校发展。深化中小学课程、教育教学方法改革，完善学生综合素质评价方案，切实搞好社会大课堂建设，加强未成年人思想道德建设，全面推进素质教育。加强农村教师队伍建设，推进学区化管理、教师交流等改革试点，促进优质教育资源均衡配置。加大对特殊教育的政策倾斜和扶持力度。完善学前教育办学体制，改扩建30所公办幼儿园，改善乡镇中心幼儿园办园条件，新增1.2万个入园名额。加强职业教育示范学校和实训基地建设，推动组建职业教育集团，扩大高职自主招生改革试点。深入推进高等教育内涵式发展，做好大学生思想政治教育工作，支持高校承担重大科技项目，优化专业和学科结构，加快特色建设步伐。

　　推进医疗卫生事业发展。整合公共卫生服务资源，提高公共卫生管理和服务水平。深入开展“健康北京人”行动，进一步普及健康知识，提高市民健康水平。完善农村三级卫生体系，完成远郊区县医疗中心、村卫生室、健康工作室建设。健全社区卫生服务机构补偿和人员绩效考核机制，进一步充实基层医务人员队伍，提高基层卫生服务水平。积极发展中医药事业，在全市公立二级以上综合医院设立中医临床科室和中药房。在基层医疗卫生机构落实基本药物制度，实行全市统一招标、统一配送、同城同价。切实加强甲型H1N1流感等传染病防控，提高突发公共卫生事件应急处置能力。

　　发展文化体育事业。继续实施文化惠民工程，全面完成街道、社区文化中心建设任务，扩大图书馆、博物馆向公众免费开放范围，新建1020个农家书屋。研究制定服务规范和标准，提升基层公共文化设施服务水平。加强互联网、手机等新媒体服务管理的制度建设，实施高清交互数字电视应用工程，支持电信、邮政事业创新发展。进一步繁荣哲学社会科学，大力发展文学艺术、广播电视、新闻出版事业。支持培育一批文艺演出活动精品。继续加强历史文化名城保护，启动一批文物修缮工程，促进非物质文化遗产保护利用。加大体育生活化社区创建力度，促进体育与生活融合。继续做好奥运场馆赛后利用工作，积极吸引国际体育机构，提升重大赛事举办水平，加快奥林匹克中心区等体育产业功能区发展。

　　高度重视住房保障。坚持结构调整，切实加大住房保障力度，发挥市场配置资源的基础作用，规范发展二手房市场和房屋租赁市场，促进首都房地产市场平稳健康发展。加快推进保障性安居工程，力争再用两年时间，全面完成三区三片棚户区改造和中心城区危旧房屋保护性修缮任务。加强老旧楼房公共设备设施安全维护。加快中小户型的廉租房、经济适用房、限价商品房、公共租赁房建设，确保占全市住宅供地面积的50%以上，确保占全市住宅新开工建设套数的50%以上，确保完成新开工建设和收购13.4万套、竣工交用4.6万套。创新政策性住房建设机制，发挥国有企业的积极性，广泛吸引社会资金参与。鼓励集体经济组织依照规划、利用存量建设用地发展公共租赁房。加强政策性住房安全质量和配租配售管理，大力推进优质公共资源优化配置，努力让群众住得放心、住得满意。继续整顿和规范房地产市场秩序，依法查处捂盘惜售、占地不用、哄抬房价等违法违规行为，遏制房价过快上涨势头。

　　(六)深入推进改革开放。

　　全面适应国际国内形势发展变化要求，继续深化重点领域和关键环节改革，拓展对外开放的广度和深度。

　　深化行政审批制度改革。充实完善市级行政服务大厅，集合各部门审批职能，为企业和社会提供更加全面、便捷的服务。抓好综合服务平台建设，实现绿色审批通道机制常态化。坚持简政放权，深化各部门内部审批改革，建立健全各项制度，加强绩效管理和效能监察。

　　深化国有企业改革。加大国有企业兼并重组力度，做实一批集团公司，推进一级企业上市融资和整体上市，大力培育具有核心竞争力的千亿元级大企业。完善国资监管体系，认真做好向企业委派董事长和财务总监工作，扩大国有资本经营预算制度试点。

　　深化社会事业改革。落实国务院医药卫生体制改革意见，坚持管理与改革并重，稳步推动公立医院改革，深化社区卫生体制改革，努力让广大群众看得上病、看得起病、看得好病。深化公益性文化事业单位管理体制改革，继续推进经营性文化事业单位转企改制。做好事业单位绩效工资改革各项工作。

　　不断提高开放型经济发展水平。进一步优化引资结构，着力引进现代服务业、高端制造业项目，大力吸引跨国公司地区总部和大型民营企业总部，推动在京外资企业增资。落实稳定外需政策，支持企业海外营销，扩大双自主产品出口。建立服务贸易工作协调促进机制，引导服务外包向高端方向发展。支持有实力的优势企业开展对外投资和跨国经营。积极吸引国际高端会议、展览和大型活动在京举办，争取国际组织在京设立机构，不断提升首都国际化水平。

　　扎实推进对口支援和区域合作。全面完成援建什邡的各项任务，认真做好对口支援拉萨、和田等地区的工作。进一步创新机制，加强与周边地区在水资源保护、生态建设、产业发展等方面的合作，实现优势互补、共同发展。全力做好参与上海世博会各项工作，支持办好广州亚运会。

　　(七)全力保持首都和谐稳定的良好局面。

　　深入开展“爱首都、讲文明、树新风”活动和群众性精神文明创建活动，大力推动社会公德、职业道德、家庭美德、个人品德建设，积极开展“六大文明引导行动”，进一步提高市民文明素质和城市文明程度。完善社区多元治理体系，健全社区参与机制，畅通居民自治渠道。依法组织第八届村民委员会选举，认真开展村务公开民主管理示范单位创建活动，加强农村基层民主建设。深入开展“五五”普法，增强全社会的法制观念和公民意识。做好社区矫正工作。加强法律援助，建设100个乡镇公益法律服务中心，实现农村公益法律服务体系全覆盖。

　　提高社会建设和管理水平。完善“枢纽型”社会组织工作体系，积极培育社会组织。健全社区建设体制，依托社区服务站，整合各部门管理力量，完善统筹协调机制，提高基层综合服务管理能力。积极推进社区规范化建设，切实解决好社区办公和服务用房问题，逐步提高社区工作者待遇。加快建设街道、乡镇社区服务中心和农村社区服务站，推广建设“一刻钟便民生活圈”试点。加强社会工作人才队伍建设，培育扶持专业社工机构，开展购买社会工作岗位试点，继续做好商务楼宇社会工作站建设。发展专业化志愿者队伍，积极开展常态化的志愿服务。

　　全面贯彻党的民族宗教政策，加快推动少数民族乡村经济发展，进一步推进宗教房产落实工作。发挥合作交流机制作用，促进京港、京澳、京台多领域合作，鼓励华人华侨参与首都建设。认真落实人口计生工作各项政策，做好流动人口计划生育服务管理。切实维护妇女和未成年人权益。加强应对人口老龄化的政策措施研究，促进老龄事业健康发展。完善助残服务体系，丰富残疾人精神文化生活，发展残疾人事业。加强国防教育和后备力量建设，深入开展拥军优属、拥政爱民和军民共建活动，做好军队干部转业安置和退役士兵就业服务，支持驻京部队建设。

　　更加自觉、更加主动地落实稳定是硬任务、是第一责任的要求，切实抓好维护首都社会稳定工作。按照抓源头、清积案、建机制、强基层的要求，做好重大决策风险评估，建立矛盾化解联动机制，积极预防、妥善处置各类群体性事件。健全安全生产问责制度，创新监管方式，加大交通运输、建筑施工、有限空间作业、地下经营空间、危险化学品、人员密集场所等重点行业和领域监管力度，有效防范和坚决遏制重特大安全事故。完善首都社会治安防控体系，加强科技创安工程建设，大力整治社会治安突出问题，依法严厉打击严重刑事犯罪，不断增强人民群众的安全感，切实维护首都和谐稳定。

　　各位代表，在新的一年里，我们将认真落实市委第三次人大工作会议精神，坚决执行市人大及其常委会的决议和决定，自觉接受人大法律监督和工作监督。认真听取各民主党派、各人民团体和各界人士的意见建议，自觉接受人民政协的民主监督。坚持与人大代表、政协委员的联系制度，健全密切联系群众的工作制度，进一步提高科学决策、民主决策水平和依法行政能力，加快建设服务型政府。一是扎实开展作风建设年活动。大力弘扬北京奥运精神和国庆60周年活动形成的宝贵精神财富，巩固深入学习实践科学发展观活动成果，树立更高标准，创造性地开展工作。坚持问政于民、问需于民、问计于民，认真落实领导干部基层联系点制度，加强改进信访工作，不断拓宽社情民意反映渠道。二是着力转变政府职能。落实政府机构改革方案，进一步优化结构、理顺关系，着力解决在具体事情上部门间职责不清、合力不够等问题。深入推进政务公开，完善行政权力公开透明的运行机制。认真总结《全面推进依法行政实施纲要》的落实情况，做好推进依法行政工作规划，加快建设法治政府。三是深入推进政府服务管理创新。总结推广奥运筹办、国庆60周年活动服务保障、应对国际金融危机的丰富经验，健全部门之间、部门与区县之间协调联动的工作机制。落实首问负责制、限时办结制、责任追究制，深入推行“三效一创”绩效管理，加强公务员队伍建设，努力提高政府行政效率和服务水平。四是加强廉政建设和反腐败工作。认真落实中央纪委五次全会精神，推广廉洁奥运经验，坚持廉洁办各项事业，充分发挥监察、审计等部门的监督职能，加强财政资金使用监管，严格控制公用经费和一般性开支，推进节约型政府建设。认真落实党风廉政建设责任制，扎实推进廉政风险防范管理工作，坚决查处腐败案件，努力建设为民、务实、廉洁、高效的政府。

　　各位代表，首都北京已经迈上新的历史征程，任务更加繁重，前景更加美好！让我们紧密团结在以胡锦涛同志为总书记的党中央周围，高举中国特色社会主义伟大旗帜，以邓小平理论和“三个代表”重要思想为指导，深入贯彻落实科学发展观，加快“人文北京、科技北京、绿色北京”建设，全力推动首都经济社会又好又快发展，为建设繁荣、文明、和谐、宜居的首善之区而努力奋斗！
